# Nutritional Disturbances of Infancy: Their Management

**Published:** 1914-11

**Authors:** 


**Nutritional Disturbances of Infancy: Their Management.** by S. Feldstein of Brooklyn, N. Y., in the Long Island Medical Journal for June, 1914. This is the best paper published in this country on this subject in a long time and fills a want long felt by the pediatrician; it is thoroughly up-to-date and gives the very latest work of the German school from personal observation and careful unbiased study. It gives the explanation of Finkelstein's classification of the digestive disturbances and in the part on their management it gives the modes of preparing the different remedial foods as well as their quantities. In short it is a masterpiece as a short monograph on a very difficult subject.—C. G. L-W.

